# Multilocus sequence typing of a global collection of *Pasteurella multocida *isolates from cattle and other host species demonstrates niche association

**DOI:** 10.1186/1471-2180-11-115

**Published:** 2011-05-25

**Authors:** Emily J Hotchkiss, J Christopher Hodgson, F Alex Lainson, Ruth N Zadoks

**Affiliations:** 1 Moredun Research Institute, Bush Loan, Penicuik, EH26 0PZ, UK

## Abstract

**Background:**

*Pasteurella multocida *causes disease in many host species throughout the world. In bovids, it contributes to bovine respiratory disease (BRD) and causes haemorrhagic septicaemia (HS). Previous studies have suggested that BRD-associated *P. multocida *isolates are of limited diversity. A multilocus sequence typing (MLST) scheme for *P. multocida *was used to determine whether the low levels of diversity reported are due to the limited discriminatory power of the typing method used, restricted sample selection or true niche association. Bovine respiratory isolates of *P. multocida *(n = 133) from the UK, the USA and France, collected between 1984 and 2008 from both healthy and clinically affected animals, were typed using MLST. Isolates of *P. multocida *from cases of HS, isolates from other host species and data from the MLST database were used as comparison.

**Results:**

Bovine respiratory isolates were found to be clonal (I^S^_A _0.45) with 105/128 belonging to clonal complex 13 (CC13). HS isolates were not related to bovine respiratory isolates. Of the host species studied, the majority had their own unique sequence types (STs), with few STs being shared across host species, although there was some cross over between porcine and bovine respiratory isolates. Avian, ovine and porcine isolates showed greater levels of diversity compared to cattle respiratory isolates, despite more limited geographic origins.

**Conclusions:**

The homogeneity of STs of bovine respiratory *P. multocida *observed, and the differences between these and *P. multocida *subpopulations from bovine non-respiratory isolates and non-bovine hosts may indicate niche association.

## Background

*Pasteurella multocida *is a Gram-negative bacterium that causes a wide range of clinical presentations in a wide range of host species [1]. It has been shown to cause respiratory disease in many animals, including cattle [2], sheep [3] and pigs [4,5] although it is also found in the respiratory tract of apparently healthy animals [6]. The organism also causes haemorrhagic septicaemia (HS) in bovids, mainly in South and Southeast Asia and sub-Saharan Africa [7]. In pigs *P. multocida *contributes to atrophic rhinitis [4] and in rabbits the organism is associated with a syndrome called "snuffles" [8]. Fowl cholera in avian species is a source of great economic losses in commercial poultry flocks and also affects wild birds [9]. In humans, *P. multocida *infections are mainly associated with animal bites [10,11].

Historically, phenotypic methods have been used to differentiate strains and it has been shown that different serotypes are associated with different hosts and clinical presentations [12]. However the usefulness of phenotypic methods is limited due to the lack of discriminatory power and the fact that they do not reflect population structure [13]. Multilocus sequence typing (MLST) provides a standardised system of typing by sequence analysis of several housekeeping genes, allowing strains to be compared worldwide and the relationship between isolates to be explored [14]. MLST can be used to explore the global epidemiology of an organism, for example identifying niche-associated strains (strains that are predominantly associated with a particular host or organ system) [15-17]. This information can be used to develop disease control measures, targeted towards these niche-associated strains.

An MLST scheme has recently been established for *P. multocida*, the *Pasteurella multocida *Rural Industries Research and Development Corporation (RIRDC) scheme [18,19]. This scheme was originally designed to type avian isolates and these comprise the bulk of submitted data; it has since been used by the international research community to submit data relating to several other host species. An alternative scheme, the *Pasteurella multocida *Multi-host MLST scheme [20] (hereafter referred to as "the alternative MLST scheme") is also available but at the time of data analysis it was not possible to submit isolates into this database.

*Pasteurella *isolates from avian species have high levels of diversity; there were 26 sequence types (STs) in 63 Australian avian *P. multocida *isolates and most STs (16/26) consisted of a single isolate [18]. This concurs with previous findings using non-MLST methods [13,21]. In cattle, diversity has been shown to be limited, but results were based on limited geographic regions [22,23].

We wanted to establish whether the limited diversity observed in bovine respiratory isolates is indicative of niche association, rather than a reflection of a limited sample population or the method's discriminatory power. Therefore we used the published (RIRDC) MLST scheme to type a global collection of isolates and to compare results across host species, clinical manifestations and geographic origins.

## Results

Complete results are available for 195 *P. multocida *isolates, as one avian and five cattle respiratory isolates failed to amplify at 1 of 7 loci after repeated attempts. Primer set ZWF-F1/ZWF-R1 failed to amplify 3 isolates; these were successfully amplified and sequenced using ZWF-F2/ZWF-R2 (all three isolates were allele *zwf-1*).

Each locus had between 16 and 26 alleles and the proportion of polymorphic sites varied from 4.6% (*mdh*) to 13.1% (*est*) (mean of 7.2%) (Table 1). The dN/dS ratios at all loci were less than 1, indicating that genes used were not under selective pressure.

**Table 1:** Characteristics of the loci used in *Pasteurella multocida *RIRDC MLST scheme, when applied to 195 isolates of diverse origin.

	Allele Length (bp)	No. of alleles	% Polymorphic sites	dN/dS
*adk*	466	16	5.8	0.076

*est*	536	26	13.1	0.23

*pmi*	602	24	5.3	0.15

*zwf*	500	25	8.8	0.017

*mdh*	521	17	4.6	0.089

*gdh*	530	16	8.3	0.059

*pgi*	560	24	5.0	0.020

**Figure 1:**
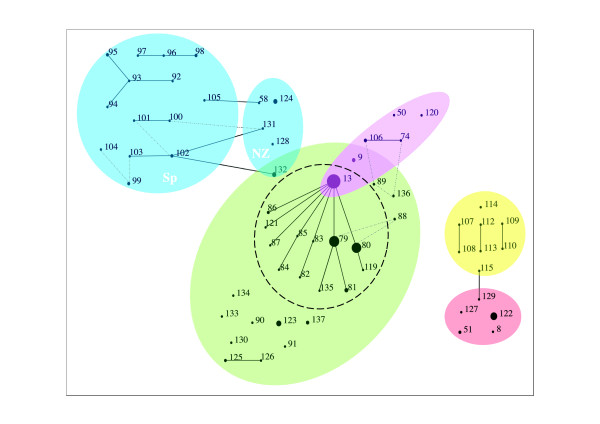
**Relationship between host species and sequence type in *Pasteurella multocida *isolates after multilocus sequence typing**. eBURST analysis of *Pasteurella multocida *isolates typed in the current study (n = 195). Outlined in blue are ovine isolates (Sp = Spanish, NZ = New Zealand), in purple are porcine isolates, in yellow avian isolates, green are bovine respiratory isolates and pink are isolates from tropics (bovine non-respiratory isolates and 2 elephant isolates). The dashed circle encloses clonal complex 13 (CC13). Grey dashed lines connect dual locus variants.

A total of 62 STs were assigned to the 195 *P. multocida *isolates analysed. Where members of a group were defined as sharing 6 of 7 alleles, eBURST divided the isolates into 22 singletons and 12 groups (either pairs of single locus variants or larger groupings of related STs) (Figure 1). Data were also explored using less stringent criteria for eBURST group definition (5 of 7 alleles shared alleles), allowing for inclusion of dual locus variants (DLVs) in groups, in the absence of single locus variants (SLVs) connecting them to the remainder of the group. In this case, the isolates divided into 11 groups and 17 singletons; there were no major changes to population structure (Figure 1).

Within cattle respiratory isolates, 105/128 belonged to clonal complex (CC) 13 (sharing 6 of 7 alleles) (Figure 1). The isolates in CC13 originated from UK, USA and France from different time periods (1984-2008) from both apparently healthy and diseased individuals. Over two thirds of bovine respiratory isolates (91 of 128) belong to just three STs (ST13, ST79 and ST80). All three of these STs included UK isolates, ST13 and ST80 included French isolates and 7 of 8 US cattle isolates were ST79 (the remaining US isolate was ST135, an SLV of ST79).

Seven isolates from calves sampled as part of a cross-sectional study in Scotland in 2008, from 7 different farms, grouped into ST123, which was unrelated to any other ST found (using the criterion of sharing 5 of 7 alleles). At the *est *locus, these isolates had a unique allele (allele *est-50*) which has a single nucleotide insertion, resulting in a frame shift mutation. The functional significance of this is unknown.

The majority of HS isolates (9 of 12; 7 cattle, 2 buffalo) belonged to a unique sequence type (ST122), which also included 2 elephant and one bison isolate of unspecified clinical status. The 28 ovine isolates grouped into 19 STs; no ST was found in both Spanish and New Zealand sheep, although multiple closely related STs (SLVs and DLVs) were identified across both groups (Figure 1). Seven porcine isolates were typed as ST13 and an additional 6 isolates belonged to 5 STs, with one ST (ST9) also found in cattle, two STs that were DLVs of cattle-associated STs and 2 STs found in pigs only (Figure 1). Eight novel STs were detected in the eight avian isolates typed in the current study.

Most STs were specific to host of origin (Figure 1), the exceptions being ST13 (40 bovine respiratory and 7 porcine isolates), ST122 (10 bovine HS and 2 elephant isolates), ST 132 (3 ovine and 1 bovine isolates) and ST9 (1 porcine, 1 bovine and 1 human isolate).

**Figure 2:**
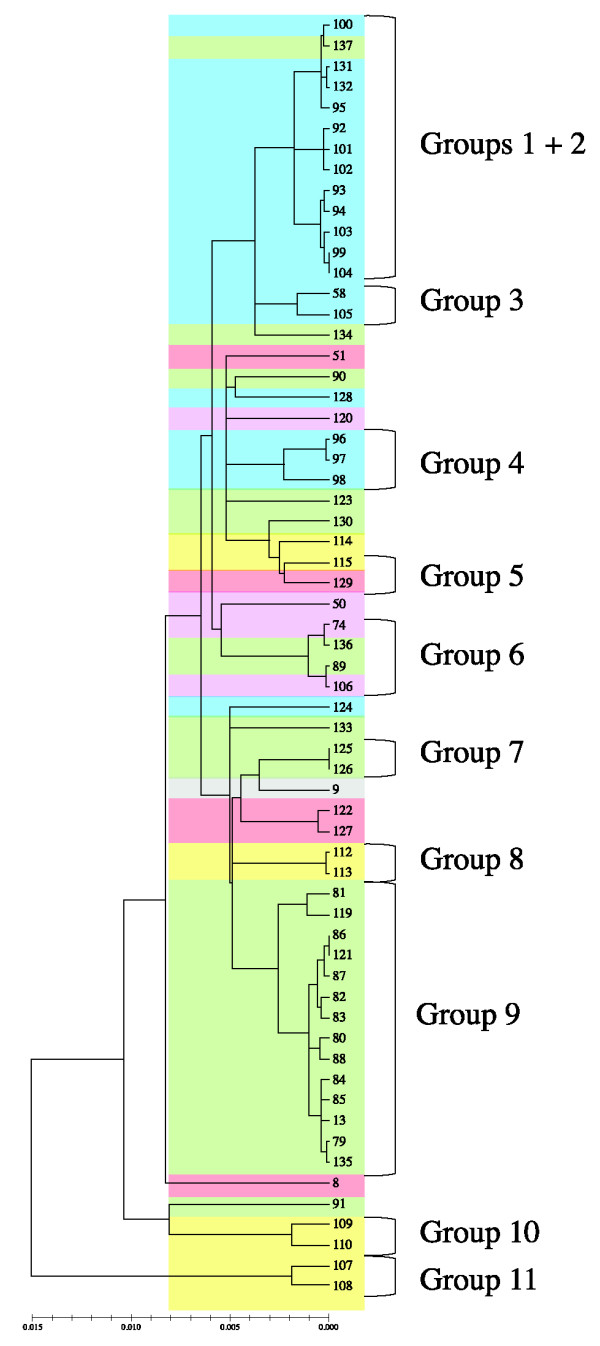
**Genetic relationships between the sequence types of *Pasteurella multocida *identified in this study**. Neighbour Joining phylogenetic tree based on concatenated sequences of 62 sequence types of *P. multocida*, showing main host association (blue = ovine isolates, purple = porcine isolates, yellow = avian isolates, green = bovine respiratory isolates, pink = bovine non-respiratory isolates and 2 elephant isolates, grey = no clear host association). Eleven groups, defined as isolates sharing 5 of 7 alleles, are shown.

A highly significant Standardised Index of Association (I^S^_A_) (0.45, P = 0.000) in cattle respiratory isolates indicated the presence of linkage disequilibrium within this population of *P. multocida *isolates and the results of SplitsTree analysis corroborated this, showing a tree-like, rather than a network, structure (Additional file 1, Figure S1). Significant linkage disequilibrium was also detected when all 195 isolates were analysed (I^S^_A _= 0.33, P = 0.000) and a tree-like structure was again observed on split decomposition analysis (Additional file 2, Figure S2). In the absence of strong evidence for recombination, a Neighbour-Joining tree was constructed from concatenated sequences (Figure 2). The population structure as demonstrated by eBURST analysis was generally maintained, with some substructuring within populations associated with specific niches, for example within ovine and bovine respiratory isolates. Bovine respiratory isolates identified as CC13 formed a discrete cluster with the inclusion of ST88, which is a DLV of STs 79 and 80; no bovine non-respiratory associated ST was related to this cluster (Figure 2). The tree divided the isolates into clusters which corresponded with the groups identified when eBURST classified a group as sharing 5 of 7 alleles.

### Comparison with isolates already submitted to the MLST database

At the time of submission, the *P. multocida *(RIRDC) database was comprised mainly of avian isolates (135 of 185) as well as 24 porcine and 5 bovine isolates. Fowl cholera was the main recorded disease (81 isolates); respiratory disease or pneumonia was recorded in 18 submissions. The isolates were mainly Australian in origin (n = 80), with 7 isolates originating from the UK. The isolates in the database represented 78 STs.

The current study produced 47 new alleles and 55 new allelic profiles (STs). Only 7 STs were already in the database (ST8, ST9, ST13, ST50, ST51, ST58 and ST74).

The database included 11 isolates in CC13, including isolates belonging to ST13 (6 pig, 2 turkey and 1 cattle isolate), ST70 (1 pig isolate) and ST44 (1 turkey isolate). These findings, coupled with results from the current study, indicate that CC13 is associated strongly, but not exclusively, with bovine respiratory isolates with porcine isolates commonly included in this clonal complex.

Results of the current study are consistent with data already submitted to the database in many instances. For example, ST122, identified in the current study as being associated with isolates from cases of HS, is an SLV of ST63 from the database, which represents a buffalo isolate (albeit of unknown geographic and clinical origin). Two of the STs identified in avian species in the current study (ST-109 and ST-110) are SLVs of ST40 from the database, which also represents avian isolates (chicken and herring gull).

Combined analysis of isolates from the current study and those already in the database at the time of manuscript preparation showed that of 137 STs, 95 were comprised of just one isolate. Of the remaining 42 STs, 3 were lacking sufficient data to determine host association. Nine STs were found in more than one host although the majority of these showed evidence of a predominant association with one host type (Table 2). For example ST13 appears to be bovine associated but not bovine specific, and the same is true for STs 5, 8 and 37 in avian species and ST50 in pigs. STs 9 and 58 are notable exceptions - to date ST9 has been detected in isolates of avian, bovine, porcine and human origin. Thirty STs are represented by multiple isolates and have so far only been identified in one host type, although in some cases this may be explained by collection of epidemiologically related isolates.

**Table 2:** Host association with sequence type (ST) of *Pasteurella multocida *isolates typed by multilocus sequence typing

Host association	Host specific	ST	Avian	Porcine	Ovine	Bovine	Other
		**5**	3				1 (mouse)
	**No**	**8**	10			1	
		**37**	6				1 (rabbit)
	
		**1**	5				
		**2**	13				
		**3**	3				
		**7**	5				
**Avian**		**12**	3				
		**16**	2				
	**Yes**	**20**	9				
		**30**	2				
		**31**	2				
		**34**	2				
		**35**	13				
		**39**	2				
		**40**	2				

	**No**	**13**	2	13		41	
		**122**				10	2 (elephant)
	
		**51**				3	
		**79**				27	
**Bovine**		**80**				24	
	**Yes**	**81**				4	
		**86**				2	
		**123**				7	
		**125**				2	
		**137**				3	

	**No**	**50**	1	9			
	
		**73**		2			
**Porcine**	**Yes**	**74**		2			
		**106**		2			

	**No**	**132**			3	1	
	
		**95**			2		
**Ovine**		**98**			2		
	**Yes**	**99**			2		
		**102**			2		
		**124**			4		

**None**	**No**	**9**	4	2		1	1 (human)
		**58**	1	1	1		

Included are isolates typed in the current study and isolates deposited in the *P. multocida *RIRDC MLST database, where relevant data were available.

## Discussion

The focus of the current study was cattle respiratory isolates, which we have found to be predominantly clonal, belonging mainly to CC13. The isolates in CC13 include cattle isolates from a range of countries, years and presentations. Preliminary studies had suggested clonality among bovine respiratory *P. multocida *isolates [22,23] but clonality of cattle isolates cannot be confirmed in isolation; if a typing mechanism indicates clonality but no other host species are examined, it is not clear whether the isolates are truly clonal or if the typing scheme is not appropriate for the organism. In this case, the fact that the scheme clearly differentiates *P. multocida *isolates within and between host species, and differentiates bovine respiratory and non-respiratory isolates, suggests that the findings in cattle are robust.

MLST (often in conjunction with other typing methods) has been used to determine host or niche association in many pathogens, for example to explore zoonotic potential of animal pathogens, to support source attribution for human infections and to identify host or niche specific clones that can be investigated in depth to understand host adaptation and host-pathogen interactions. MLST of *Campylobacter jejuni *has identified poultry-associated strains as the major cause of foodborne infection [24,25]. In contrast, other strains of *C. jejuni*, for example from the environment and wild birds, are not associated with disease in humans [25]. For *C. jejuni*, as for *P. multocida*, host-association transcends geographic boundaries [17]. Similar phenomena are observed in Gram-positive species, e.g. *Staphylococcus aureus*, which is a common cause of disease in humans and ruminants. MLST has identified clonal complexes of *S. aureus *that are associated with humans, cattle and small ruminants, respectively, and these clonal complexes are found across multiple continents [26,27]. Further study of host-associated strains has led to identification of molecular correlates of host specialisation in *Campylobacter *[28] and *S. aureus *[29] and our findings could form the basis for similar work in *P. multocida*. Within many bacterial species, generalist strains also exist. Examples would include *C. jejuni *ST45 [25], *S. aureus *ST398 [30] and *P. multocida *ST9 from the current study.

Whilst the majority of bovine respiratory isolates did group into CC13, there were a number that did not. The epidemiological significance of these outliers is unknown; isolates were from clinically and non-clinically affected animals in the UK and France and were collected over a number of years. Strains of other pathogens that appear unrelated by MLST and other molecular analyses (but may share other common characteristics) have been shown to cause the same clinical picture in the same host species, for example *S. aureus *in bovine mastitis [15].

Isolates from both clinically affected and apparently healthy animals grouped together in CC13. As housekeeping genes were used, this is perhaps not surprising as virulence is likely to be driven by other genetic markers, for example those encoding outer membrane proteins (OMPs), iron acquisition factors and colonisation factors [31,32]. In addition, there may be other non-pathogen related drivers of disease, such as host immunity. For example, the ovine isolates identified here as NZ originated from sheep being exported by sea when an outbreak of pneumonia caused a number of fatalities [33]. Multiple serotypes of *P. multocida *were identified as the primary pathogen in necropsied sheep, suggesting that diverse commensal flora in the respiratory tract of the sheep behaved as opportunistic pathogens when the sheep encountered stress and adverse environmental conditions. In the current study, multiple STs were also detected in this outbreak but MLST has been shown to lack sufficient discriminatory power when used at farm level in cattle [23]. In cattle, more discriminatory typing methods should be employed where local epidemiology is being studied (for example outbreak investigations). In these cases, methods such as RAPD and PFGE may be appropriate tools [23]. OMP profiling has also been shown to be more discriminatory than MLST in *P. multocida *isolates [22].

HS isolates were distinct from bovine respiratory isolates, suggesting that isolates in CC13 are not just cattle associated, but more specifically associated with the bovine respiratory tract niche. However it is also possible that there has been geographical substructuring or ecological isolation of populations - we do not have access to bovine respiratory tract isolates from the Tropics or HS isolates from Europe/USA to test this theory. HS isolates have been shown to differ from respiratory isolates at phenotypic markers, particularly capsular serotypes (HS isolates are usually capsular group B whereas bovine respiratory isolates are usually capsular group A) [2,7].

The alternative MLST scheme has also found cattle samples to be clonal in nature [22], with 22 of 32 bovine respiratory isolates grouping into one clonal complex which also included 11 porcine isolates. In the alternative scheme, HS isolates were not related to bovine respiratory isolates, using the criterion of sharing 5 of 7 alleles and data were consistent with the RIRDC scheme in that some STs were non-host specific whereas others appeared to be host associated. One of the major advantages of MLST is the portability of methods and results, which is why we chose to use the (RIRDC) scheme rather than the alternative scheme. Because results are portable and standardised, they can be compared across database entries from multiple contributors. When attempts were made to use the database to explore host association of STs, however, it was not always easy to determine whether STs that appeared host specific could reflect epidemiologically linked isolates. For example, ST2 appears to be host specific, comprising 13 isolates, all of avian origin. Examination of an associated reference reveals that 12 of these isolates are epidemiologically related [18]. The epidemiological value of data from MLST databases is limited by the isolates and data submitted by contributors. Where contributors only submit data for one representative isolate per ST, epidemiological interpretations may be misleading [34]. With expansion of an MLST scheme, referring to all associated publications to determine, for example, frequency of occurrence of STs or epidemiological relatedness of isolates becomes less feasible.

## Conclusions

The analysis by MLST of this global collection of isolates from multiple host species and disease syndromes has identified niche association in bovine respiratory *P. multocida *isolates. Development of an efficacious vaccine against *P. multocida *would be a valuable tool in reducing the significant economic losses, and welfare concerns, associated with BRD. Future work in this area should target the dominant, niche-associated strains such as those included in CC13.

## Methods

The aim of sample selection was to include as diverse a range of isolates as possible, from different host species, clinical presentations, geographical locations and years of collection. As they were of particular interest, the majority of isolates were obtained from cattle (Table 3). These isolates were drawn from 6 collections, 3 continents and from healthy as well as diseased animals (bovine respiratory disease and HS). Isolates from other host species (Table 3) and data from the MLST database were used for comparison.

**Table 3:** Summary of sources of *P. multocida *isolates selected for analysis by multilocus sequence typing.

Host	n	Source	Year	Epidemiological or Clinical Data	Reference
**Bovine respiratory**	37	Scotland	2008	Cross-sectional survey. 1 isolate per RAPD type per farm (32 farms), predominantly non-clinical	[6]
	30	UK	1984-2007	Epidemiologically unrelated. Clinical.	
	19	UK	2000	Single BRD outbreak (clinically affected and unaffected)	
	8	USA		Feedlot cattle	
	39	France	2008	BRD outbreaks on farm. 1 isolate per RAPD type per farm (20 farms)	

**Bovine non-respiratory**	12	Southeast/South Asia		Haemorrhagic septicaemia (HS)	
	3	Tropics		Clinical status unknown. Grouped with HS on basis of isolate origin	

**Ovine**	10	NZ		Multiple source farms, outbreak during transport	[33]
	18	Spain		Clinical, several farms within one region	

**Porcine**	13	UK		Bronchopneumonia. Distinct PFGE types	[5]

**Avian**	9	Southeast Asia/unknown		Fowl cholera	

**Other**	3	Various		2 elephants (Asia), 1 human	

***Total***	201				

RAPD: random-amplified polymorphic DNA; BRD: bovine respiratory disease; PFGE: pulsed-field gel electrophoresis

Stocks of 201 *P. multocida *isolates stored previously at -70°C in glycerol were cultured overnight on sheep blood agar (5% citrated sheep blood in agar No.2 base; E&O Laboratories Ltd), at 37°C. Colonies were suspended in 500 ul sterile water, vortexed and heated at 95°C for 10 minutes. These lysates were used as template in a PCR to confirm species, based on the *kmt *gene [35].

The DNA was used to amplify loci from 7 housekeeping genes. The primers and conditions were as per the MLST (RIRDC) scheme [18,19] As specified, 7 loci (*adk*, *est*, *pmi*, *pgi*, *zwf*, *gdh*, *mdh*) were used and gene fragments of lengths 570-808 bp were amplified. For the *zwf *locus, both sets of primers were used on all samples (ZWF-F1/ZWF-R1 and ZWF-F2/ZWF-R2). After confirmation of amplification by gel electrophoresis, PCR product was purified and sequenced in both directions by a commercial company (GATC Biotech). Forward and reverse sequences were aligned and manually inspected using SeqMan (DNASTAR Lasergene 8). Consensus sequences were stored in FASTA format. High quality double stranded DNA was used to assign alleles, with lengths ranging from 466 to 602 bp (Table 1). At each locus sequences were checked for existing alleles using the MLST database. New alleles and STs were assigned by the MLST database curator, after verification of trace files.

STs were analysed using eBURST v3 [36,37]. Groups were defined where STs shared 6 of 7, and also 5 of 7, alleles. Split decomposition analysis was performed on allelic profile data using SplitsTree v4 [38,39] and the standardized index of association (I^S^_A_) was calculated, both for cattle respiratory isolates alone and for all isolates using LIAN v3.5 [38,40]; the Monte-Carlo method with 1000 samplings was used to determine significance. Only one representative of each allelic profile was included. A Neighbour Joining tree was constructed from the concatenated sequences (3715 bp) using the Jukes Cantor algorithm with 1500 bootstrap replicates (MEGA v.5.03) [41]. The number of polymorphic sites, allelic frequencies and ratio of nonsynonymous to synonymous substitutions (dN/dS) was calculated for all loci using START v2 [42].

## Authors' contributions

EJH, JCH and RNZ participated in the design of the study. EJH carried out the laboratory work and sequence analysis and drafted the manuscript. JCH coordinated and maintained the isolate collection and edited the manuscript. FAL established typed collections of UK porcine isolates and Asian bovine isolates and metadata. RNZ conceived of the study and edited the manuscript. All authors read and approved the final manuscript.

## Supplementary Material

Additional file 1 **Figure S1 Split decomposition analysis performed on 27 sequence types identified in 128 bovine respiratory *Pasteurella multocida *isolates**. Click here for file

Additional file 2 **Figure S2 Split decomposition analysis performed on 62 sequence types identified in 195 *Pasteurella multocida *isolates, from different host species and disease syndromes**. Click here for file
