# Supplementary figures and images for: Multilocus sequence typing of a global collection of Pasteurella multocida isolates from cattle and other host species demonstrates niche association
Source: BMC Microbiol. 2011 May 25;11:115. doi: 10.1186/1471-2180-11-115 (PMC3120644; doi:10.1186/1471-2180-11-115)

0.1

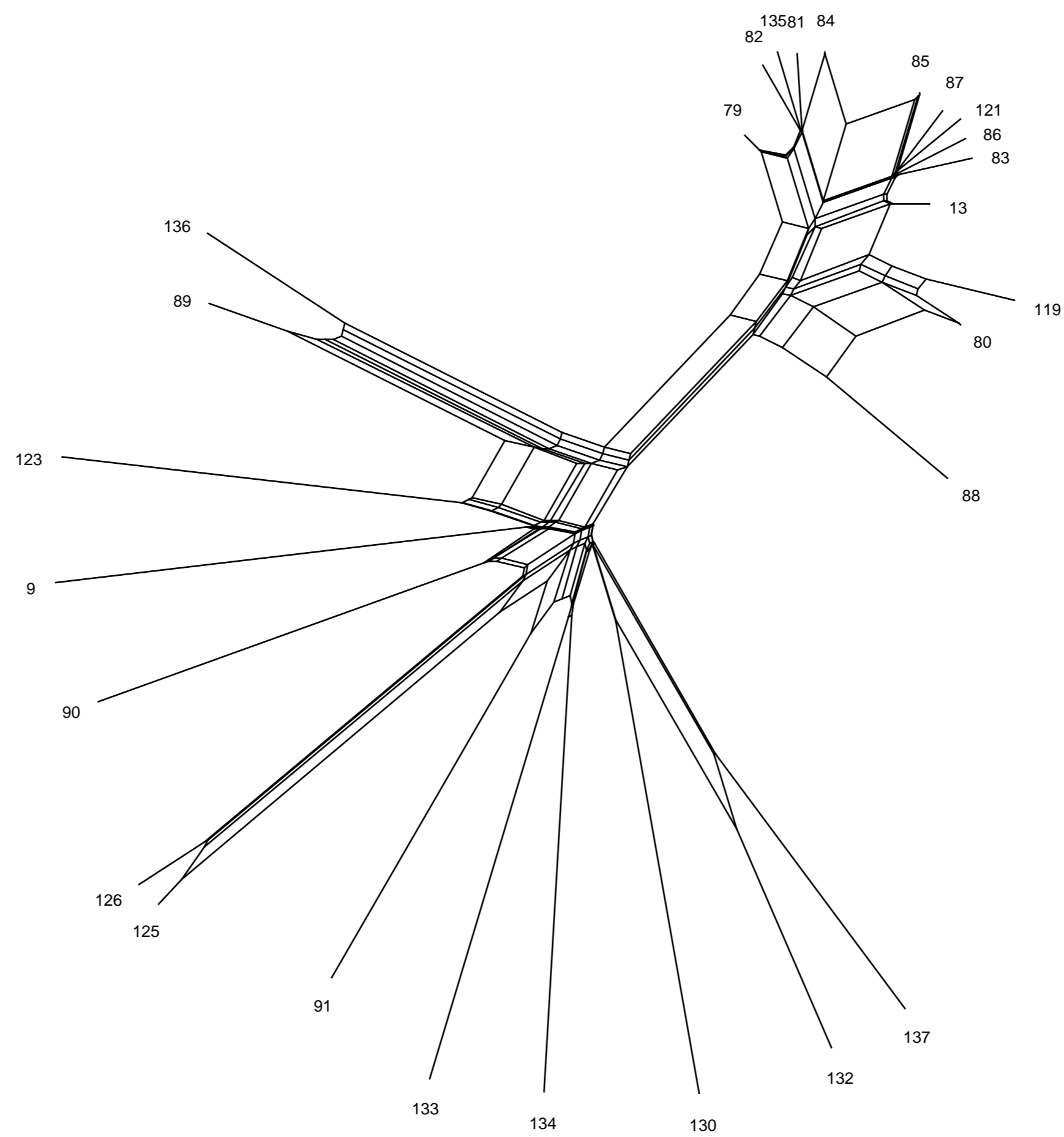

Supplement: Additional file 1 — Figure S1 Split decomposition analysis performed on 27 sequence types identified in 128 bovine respiratory Pasteurella multocida isolates. [file 1471-2180-11-115-S1.PDF]

0.1

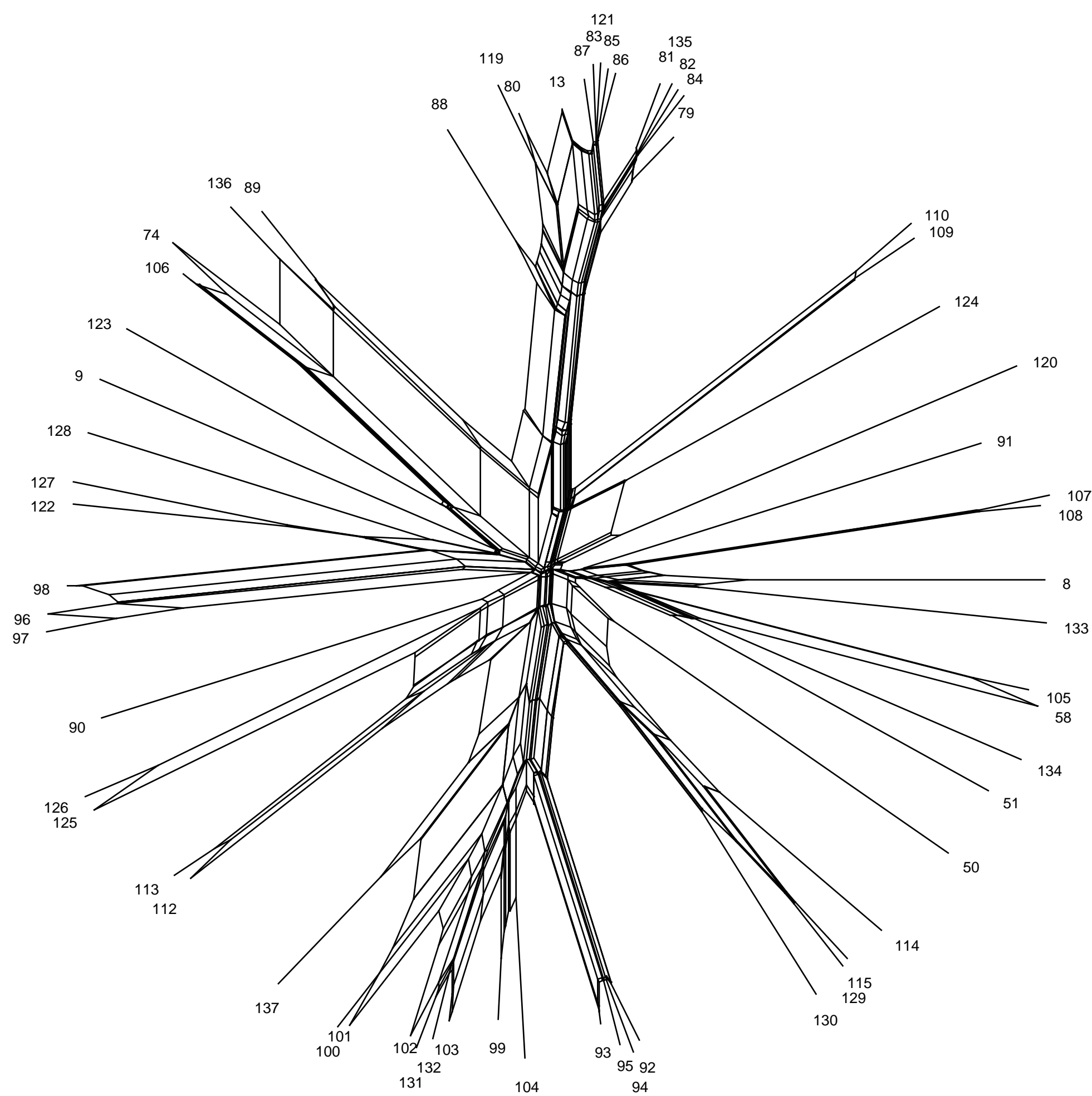

Supplement: Additional file 2 — Figure S2 Split decomposition analysis performed on 62 sequence types identified in 195 Pasteurella multocida isolates, from different host species and disease syndromes. [file 1471-2180-11-115-S2.PDF]
